# Improving data interpretability with new differential sample variance gene set tests

**DOI:** 10.21203/rs.3.rs-4888767/v1

**Published:** 2024-09-09

**Authors:** Yasir Rahmatallah, Galina Glazko

**Affiliations:** 1 Department of Biomedical Informatics, University of Arkansas for Medical Sciences, Little Rock, AR 72205, USA.

**Keywords:** Gene set analysis, differential variability, minimum spanning tree, Anderson-Darling, Cramer-Von Mises

## Abstract

**Background:**

Gene set analysis methods have played a major role in generating biological interpretations from omics data such as gene expression datasets. However, most methods focus on detecting homogenous pattern changes in mean expression and methods detecting pattern changes in variance remain poorly explored. While a few studies attempted to use gene-level variance analysis, such approach remains under-utilized. When comparing two phenotypes, gene sets with distinct changes in subgroups under one phenotype are overlooked by available methods although they reflect meaningful biological differences between two phenotypes. Multivariate sample-level variance analysis methods are needed to detect such pattern changes.

**Results:**

We use ranking schemes based on minimum spanning tree to generalize the Cramer-Von Mises and Anderson-Darling univariate statistics into multivariate gene set analysis methods to detect differential sample variance or mean. We characterize these methods in addition to two methods developed earlier using simulation results with different parameters. We apply the developed methods to microarray gene expression dataset of prednisolone-resistant and prednisolone-sensitive children diagnosed with B-lineage acute lymphoblastic leukemia and bulk RNA-sequencing gene expression dataset of benign hyperplastic polyps and potentially malignant sessile serrated adenoma/polyps. One or both of the two compared phenotypes in each of these datasets have distinct molecular subtypes that contribute to heterogeneous differences. Our results show that methods designed to detect differential sample variance are able to detect specific hallmark signaling pathways associated with the two compared phenotypes as documented in available literature.

**Conclusions:**

The results in this study demonstrate the usefulness of methods designed to detect differential sample variance in providing biological interpretations when biologically relevant but heterogeneous changes between two phenotypes are prevalent in specific signaling pathways. Software implementation of the developed methods is available with detailed documentation from Bioconductor package GSAR. The available methods are applicable to gene expression datasets in a normalized matrix form and could be used with other omics datasets in a normalized matrix form with available collection of feature sets.

## Background

Gene expression is a quantitative trait translating genetic changes (e.g. SNPs, frameshifts, deletions, splicing disturbing variants, chromosomal aberrations) into dynamic molecular phenotypes. For Mendelian diseases, application of gene expression analysis (RNA-seq) has increased diagnostic rate by 8–36% and improved our understanding of molecular mechanisms of the variants [1–3]. Gene expression is the most broadly studied class of molecular data for cancer so far (The Cancer Genome Atlas repository [4]) and has led to development and validation of predictive biomarkers in lung [5], breast [6, 7], colon [8, 9], and other tumor types [10–14]. Transcriptome analysis helps identify gene expression signatures that can predict response to specific cancer therapies [15, 16] and molecular correlates of metastasis [17].

The routine omics data analysis of individual genes (proteins) usually includes the analysis of Differentially Expressed (DE) genes, measuring the mean expression differences between sets of samples representing conditions of biological interests, for example disease and healthy phenotypes. However gradually, it became evident that gene expression variability is also important for understanding molecular mechanisms underlying phenotypic differences. Expression variability has been associated with biological function [18–22], ageing [23–25], is implicated in human diseases [20, 26, 27], cancer [28–31] and has diagnostic and prognostic potential in cancer [28–30, 32–34]. Moreover, there are genes with consistently higher across-sample variability in tumors of different origin as compared to normal samples [30]. These differentially variable genes (anti-profiles) can serve as a robust molecular signature for multiple cancer types [30, 33]. Notably, heterogeneity is an intrinsic property of cancer cells since the formation of tumor is dependent on the acquisition of oncogenic mutation and further clonal selection through Darwinian-like evolution. Variability itself is a cornerstone of Darwin’s evolutionary theory and a characteristic of cancer cells [35].

Genes and proteins are organized in functional units acting in concert. Phenotypic changes are associated with changes of multiple genes in biological pathways and molecular networks, more often than single gene alterations. In line with these ideas almost two decades ago a new development in DE analysis was Gene Set Analysis (GSA) technique, first implementation known as Gene Set Enrichment Analysis (GSEA) [36], followed by more than 100 GSA methods/tools [37]. The advantages of pathway analysis (or GSA) are well known but it’s worth reiterating: 1) It allows for the incorporation pre-existing biological knowledge into the analysis; 2) it employs a hypothesis-testing framework accounting for differences in various distributional statistics; and 3) it enhances the power of analysis by decreasing the number of hypotheses to test [38–41]. Gene set analysis methods differ in many aspects, such as univariate/multivariate, underlying assumptions, definition of enrichment, null hypotheses, significance assessment, but share a common feature: They are generally testing ‘average’ difference between pathway’s genes expression under different phenotypes.

Because of the accumulated evidence that initiation and progression of cancers are dependent on the acquisition of multiple driver mutations that activate oncogenic pathways and inactivate tumor suppressor pathways [35], it would be expected that there are many computational approaches allowing one to identify differential variability in gene sets. However, differential variability analysis for individual genes was not immediately followed by the development of gene set approaches. The first attempts to present GSA methods that test for differential sample variance (DV) between two phenotypes was done by our group [38], and some others [39, 40]. We demonstrated in [38] that for three different cancer types, DV approach was able to identify cancer-specific pathways, while pathways identified using differential mean approach were shared between the three cancer types. Other GSA approaches claiming to perform differential variability test, e.g. DIRAC and EVA [41], compare variability in gene ranks within a pathway between two phenotypes rather than sample variance, hence are out of the scope of our manuscript.

Here we propose to use ranking schemes based on the minimum spanning tree to generalize the Cramer-Von Mises and Anderson-Darling statistics into gene set analysis methods to detect differential sample variance or mean. We characterize the detection power and Type I error rate of these two methods with two additional methods developed based on the same principle using simulation results with different parameters. Finally, we apply the proposed methods to microarray and bulk RNA-sequencing gene expression datasets and demonstrate the biological relevance of the detected Hallmark gene sets by our differential sample variance methods.

## Methods

### Rank-based statistical methods.

**Figure 1.**
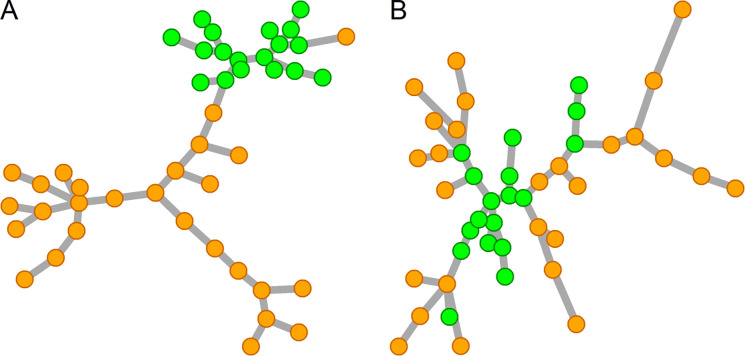
Examples of minimum spanning tree graphs: A) Two phenotypes indicted by different colors are well-separated in the tree indicating differential sample mean, B) two phenotypes show differential sample variance where samples from the green phenotypes occupy the backbone of the tree while samples from the orange phenotypes occupy the peripheries.

We introduce nonparametric GSA methods by generalizing univariate statistics into multivariate methods based on the minimum spanning tree (MST). For a gene set of size p, let $n_{1}$ and $n_{2}$ *p*-dimensional measurements respectively represent samples from phenotypes X and Y. These $N=n_{1}+n_{2}$ samples can be represented as vertices of an edge-weighted undirected graph where edge weights represent some distance measure between samples in the $R^{p}$ space, such as the Euclidean distance. The MST of this graph is the acyclic subset of edges selected from the full set of N(N-1)/2 possible edges in the full graph such that the sum of edge weights is minimal. The distance between any two vertices in the MST correlates with their distance in the $R^{p}$ space. This property allows the multivariate generalization of rank-based univariate statistical methods by assigning vertices in the MST ranks based on a specific scheme. The test statistic is calculated based on vertex ranks and significance is estimated non-parametrically using a sample permutations approach. The ranking scheme can assign a specific alternative hypothesis such as differential sample mean (DM) or differential sample variance (DV) more detection power. Figure 1 shows two examples of MST that demonstrate DM (panel A) and DV (panel B) between two phenotypes. It has been shown that the high directed preorder (HDP) ranking scheme is suitable for detecting DM $\left(\mu_{X}\neq \mu_{Y}\right)$, while the radial ranking scheme is suitable for detecting DV $\left(\sigma_{X}\neq \sigma_{Y}\right)$ [38,42]. We have applied the MST-based approach to generalize the Kolmogorov-Smirnov statistic into a gene set test to detect DM (KS) or DV (Radial Kolmogorov-Smirnov or RKS) [38]. We have also repurposed the mean deviation statistic used for GSEA to test for DM (MD) and DV (RMD) [43]. We propose here to generalize two new univariate statistics, namely the Cramer-Von Mises and Anderson-Darling, using the MST-based approach to have two new tests of DM (CVM and AD) and two new tests of DV (RCVM and RAD). The four statistics put emphasis on different regions of the deviation between two empirical cumulative distribution functions (CDFs) of two groups of ranked samples in the MST. These four statistics have the potential to capture different patterns of differential variability in gene sets. For instance, CVM assigns the middle range more detection power, while AD assigns more detection power to the tails. We also examine the effect of the MST order (K) on the methods that test for DV. When MST order K=2, we apply the radial ranking scheme to the union of the first and second MSTs. The second MST is defined as the MST of the full graph after excluding the links of the first MST. Additional MSTs can be added iteratively in a similar way for *K*>2. In total, we examine the performance of 16 options, including 4 methods that test for DM (KS, MD, CVM, and AD), and 4 methods that test for DV with K=1 (RKS1, RMD1, RCVM1, RAD1), K=2 (RKS2, RMD2, RCVM2, RAD2), and K=3 (RKS3, RMD3, RCVM3, RAD3).

### Kolmogorov-Smirnov (KS).

The KS statistic measures the maximum deviation between the CDFs of two groups of ranked samples in the MST. The KS statistic is defined as

$$KS=\sqrt{\frac{n_{1}n_{2}}{N}}\underset{i}{max}\;\left|\frac{r_{i}}{n_{1}}-\frac{s_{i}}{n_{2}}\right|$$

where $r_{i}$ and $s_{i}$ are respectively the number of samples in X and Y ranked lower than i in the MST. The MST-based multivariate generalization of the KS statistic was suggested early in a general context [42] and later implemented and characterized against DM and DV alternative hypotheses 8 in gene expression data [38, 44].

### Mean Deviation (MD).

A single-sample version of GSEA was used to estimate enrichment scores, calculated as the mean deviation between the empirical CDFs of gene ranks between a gene set of interest and the rest of the genes in the dataset [45]. We successfully repurposed this score to test if the mean deviation between the empirical CDFs of sample ranks of two groups in the MST is significant [44]. The mean deviation statistic is defined as

$$M=\sum_{i=1}^{p}\left[\frac{\sum_{j\in X,j\leq i}r_{j}^{\rho }}{\sum_{j\in X}r_{j}^{\rho }}-\sum_{j\in Y,j\leq i}\frac{1}{n_{2}}\right]$$

where $r_{j}$ is the rank of sample j in the MST, p is the gene set size, and the exponent ρ is set to 0.25 to give the ranks a modest weight.

### Cramer-Von Mises (CVM).

This statistic measures the difference between two groups of samples based on the deviation between their empirical CDFs as [46]

$$C=\frac{n_{1}n_{2}}{N^{2}}\left\{\sum_{i=1}^{n_{1}}\;\;{\left[\frac{r_{i}}{n_{1}}-i\left(\frac{1}{n_{1}}+\frac{1}{n_{2}}\right)\right]}^{2}+\sum_{j=1}^{n_{2}}\;\;{\left[\frac{s_{j}}{n_{2}}-j\left(\frac{1}{n_{1}}+\frac{1}{n_{2}}\right)\right]}^{2}\right\}$$

where $r_{i}$ and $s_{i}$ are respectively the number of samples in X and Y ranked lower than i in the MST.

### Anderson-Darling (AD).

This statistic measures the deviation between sample ranks of two groups in the MST, allowing higher weight to deviations at the tails of the distribution. The AD statistic can be defined as [47, 48]

$$A=\frac{1}{n_{1}n_{2}}\sum_{i=1}^{N-1}\;\frac{{\left(r_{i}N-i\;n_{1}\right)}^{2}}{i(N-i)}$$

where $r_{i}$ is the number of samples in X ranked lower than i in the MST. The AD statistic is a 6 weighted version of the CVM statistic.

### Significance estimation.

For KS, CVM, and AD, p-value is defined as the proportion of permutations yielding a more extreme permutation statistic ${\mathrm{stat}}_{i}$ than the observed statistic ${\mathrm{stat}}_{\mathrm{obs}}$ or

$$p-\mathrm{value}=\frac{\sum_{i=1}^{B}\;\;I\left[{\mathrm{stat}}_{i}\geq {\mathrm{stat}}_{\mathrm{obs}}\right]+1}{B\;+\;1}$$

where B is the number of permutations and I[.] is the identity function. For MD, the absolute value of the statistic is used when p-value is estimated or

$$p-\mathrm{value}=\frac{\sum_{i=1}^{B}\;I\left[\left|{\mathrm{stat}}_{i}\right|\geq \left|{\mathrm{stat}}_{\mathrm{obs}}\right|\right]\;+\;1}{B+1}$$

### Simulation setup.

We simulate gene expression under two p henotypes X and Y assuming multivariate normal distribution with different parameters. Type I error rate under the null hypothesis $\left(F_{X}=F_{Y}\right)$ and detection power under the DV $\left(\sigma_{X}\neq \sigma_{Y}\right)$ and DM $\left(\mu_{X}\neq \mu_{Y}\right)$ alternative hypotheses were estimated. To estimate Type I error rate, we generated 1000 non-overlapping gene sets of size p under groups X and Y of equal sample size, $n_{1}=n_{2}=N/2$ from the p-dimensional normal distribution $N\left(0,I_{p\times p}\right)$ where $I_{p\times p}$ is a p×p identity matrix. Type I error rate is the proportion of detected gene sets when the null hypothesis is true. Statistical significance was estimated using 1000 permutations for all methods.

To estimate detection power under different scenarios, we introduced four parameters: 1) γ, the proportion of genes in a set with true effect, 2) σ, the fold-change in variance, 3) μ the shift in mean, and 4) r the intergene correlation. Two groups of N/2 samples from p-dimensional normal distributions $N\left(0,S_{x}\right)$ and $N\left(0,\Sigma_{y}\right)$ represent two phenotypes were generated. To simulate the DV scenario $\left(\sigma_{X}\neq \sigma_{Y}\right)$, the covariance matrices $S_{x}$ and $S_{y}$ are p×p positive definite and symmetric matrices. The diagonal elements of $S_{x}$ are equal to 1 and off-diagonal elements are equal to r. Matrix $S_{y}$ is defined as

$$S_{y}=\left[\begin{array}{ll} A & B\\ C & D \end{array}\right]$$

where A is a γp×γp matrix with $A_{ij}=\sigma$ for i=j and $A_{ij}=r\sigma$ for i≠j,B and C are respectively γp×(1-γ)p and (1-γ)p×γp matrices where $B_{ij}=C_{ij}=\sqrt{\sigma }r$ for all i and j, and D is a (1-γ)p×(1-γ)p matrix with $D_{ij}=1$ for i=j and $D_{ij}=r$ for i≠j. To simulate the DM scenario $\left(\mu_{X}\neq \mu_{Y}\right)$, diagonal elements of $S_{X}$ and $S_{Y}$ are set 1 and off-diagonal elements are set to r. Since expression data is assumed to be in log-scale, the shift in mean μ is added to γp of the genes in each gene set. We generate 1000 non-overlapping gene sets. We consider the parameters γ=[0.25,0.5,0.75], r=[0.1,0.5], σ=[1,…,5], μ=[0,…,2], p=[20,60,100], and N=[20,40,60].

### Childhood B-ALL gene expression dataset.

Raw Affymetrix microarray data profiling leukemia cells in children diagnosed with B-lineage acute lymphoblastic leukemia (B-ALL) [49] was obtained from the Gene Expression Omnibus (GEO) repository [50]. Samples profiling in vitro sensitive (54 samples) and resistant (20 samples) to prednisolone patients were downloaded (GSE655 and GSE656). Samples with biased median normalized unscaled standard error (NUSE) were discarded, leaving 45 sensitive and 16 resistant samples. Raw intensities were normalized using the robust multi-array average (RMA) method and multiple probes measuring the same target mRNA were summarized based on the maximum inter-quartile range (IQR) across samples.

### Colon polyps dataset.

A subset of a colon polyps bulk RNA-seq dataset (GEO accession number GSE76987, [51]) was downloaded including 10 hyperplastic polyp (HP) and 21 sessile serrated adenoma/polyp (SSP) specimens. Preprocessing of raw reads, aligning to the hg19 human genome, and quantifying gene expression were performed following the steps in [52]. Normalized log_2_(1 + FPKM) values were used for downstream analysis.

### Gene sets.

Fifty gene sets from the Hallmark collection [53] were downloaded from the molecular signature database (MSigDB) [54]. Hallmark gene sets were derived from founder gene sets that convey specific biological state, process, or signaling pathway, and summarize the most relevant information of their original founder sets. Hallmark gene sets were selected for their biological relevance. Four prednisolone resistance gene sets from the curated collection of the MSigDB were also included to analyze the childhood B-ALL dataset. These four sets were curated from the study that generated the childhood B-ALL dataset [49] and are useful for a sanity check.

## Results

### Simulation results.

Table 1 presents the estimated significant levels for all methods at N=40,p=60, and α=0.05. Overall, all of the four statistics and MST order options (K=1,2, and 3) controlled Type I error rate at the specified significance level. Tables S1-S8 (Additional File 1) shows similar Tables for different sample size values (N=20,40, and 60) and gene set size values (p=20,60, and 100).

**Figure 2.**
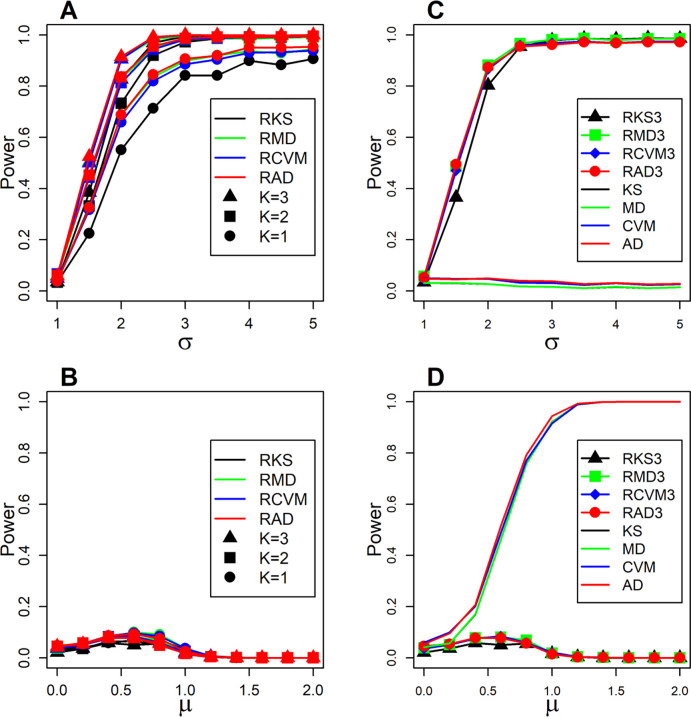
Simulated data estimates of detection power against specific alternative hypotheses. Panels A and B show the power when minimum spanning tree order K=1,2, and 3. Panel C shows high detection power achieved by methods that used the radial ranking with K=3 (RKS3, RMD3, RCVM3, and RAD3) and negligible power by methods that used the highdirected preorder ranking (KS, MD, CVM, and AD) against differential variance fold-change σ when $\sigma_{X}\neq \sigma_{Y}$. Panel D shows high detection power achieved by methods that used the high directed preorder ranking and negligible power by methods that used the radial ranking against differential mean shift μ when $\mu_{X}\neq \mu_{Y}$. Sample size N=40, gene set size p=60, proportion of genes with true difference γ=0.5, and intergene correlation r=0.1.

Figure 2 presents the detection power estimates when either the DV alternative hypothesis is is true $\left(\sigma_{X}\neq \sigma_{Y}\right)$ (Figure 2A and Figure 2C) or the DM alternative hypothesis is true ($\mu_{X}\neq \mu_{Y}$) (Figure 2B and Figure 2D) for N=40,p=60,γ=0.5, and r=0.1. For the methods that detect DV (RKS, RMD, RCVM, and RAD) the null hypothesis is rejected if they detect significant DV (p-value<0.05) and their DM counterpart methods (KS, MD, CVM, and AD) fail to detect DM (p-value≥0.05). This is done to guard against the non-negligible detection power of DV methods when $\mu_{X}\neq \mu_{Y}$. Figure 2 A shows the detection power against the variance fold-change when $\sigma_{X}\neq \sigma_{Y}$ for the four methods that use the radial ranking scheme of the MST. Gain in detection power is achieved when the MST order increases from 1 to 2 and to a lesser extent from 2 to 3. Higher MST orders yield negligible gain or none, hence the presented results consider K=1,2, and 3. Figure 2B shows a negligible detection power for all four methods regardless of the MST order (K=1,2, and 3). Similar Figures for N=[20,40,60], p=[20,60,100], γ=[0.25,0.5,0.75], r=[0.1,0.5], and K=1,2, and 3, are shown in Figures S1-S18 (Additional File 1). Figure 2C shows the detection power against the variance fold-change when $\sigma_{X}\neq \sigma_{Y}$ for the methods that detect DM or DV (K=3). Figure 2D shows the detection power against the mean shift when $\mu_{X}\neq \mu_{Y}$ for the methods that detect DM or DV (K=3). The detection power estimates for N=[20,40,60], p=[20,60,100], γ=[0.25,0.5,0.75], r=[0.1,0.5] are shown in Figures S19-S36 (Additional File 1).

### Childhood B-ALL dataset.

**Figure 3.**
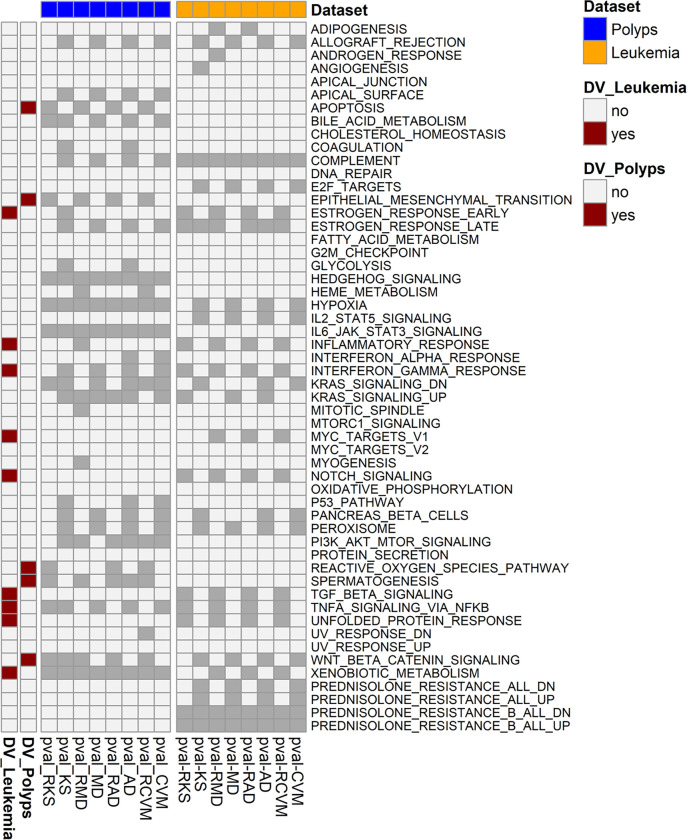
Hallmark gene sets detected by DV but not DM methods in the childhood B-ALL dataset comparing prednisolone-sensitive and prednisolone-resistant patients and in the colon polyps datasets comparing hyperplastic polys (HP) and sessile serrated adenoma/polyps (SSP). Cells with dark gray color indicate significant p-values.

Methods that detect DV or DM were applied with 10000 permutations. Gene sets were deemed having significant DV if a DV method returned significant nominal p-value (<0.05) but not the DM counterpart method (e.g. RAD but not AD). Nine Hallmark gene sets showed significant DV in the majority of the four used methods (Figure 3). Children diagnosed with B-ALL have different cytogenetic translocations that produce fusion genes with distinct molecular subtypes. We hypothesized that these subtypes will contribute to heterogeneous differences between prednisolone-sensitive and prednisolone-resistant phenotypes, and that our proposed methods that detect DV could capture such differences in relevant gene sets. Therefore, we will focus on the interpretation of gene sets detected by the majority of methods that detect DV by presenting studies from literature that support the biological relevance of these sets through association with prednisolone/glucocorticoid resistance or with B-ALL as detailed below.

Two prednisolone resistance gene sets consisting of up- and down-regulated genes in prednisolone-resistant B-ALL patients [49] were found significant by all four methods that detect DM or DV. This is expected since members of these sets are significantly different between the two phenotypes, leading to significant DM. The large difference in mean caused DV methods that show non-negligible detection power to significant DM to return small p-values. However, DV is ignored here since DV is accepted only if the methods that test DM return insignificant p-values. Another two gene sets of up- and down-regulated genes in prednisolone-resistant ALL (both B-ALL and T-ALL) patients were found significant by all DM but not DV methods. This is also expected since only a subset of these sets is up or down-regulated in the analyzed B-ALL dataset. Half of the genes were common between the two up-regulated gene sets in ALL and B-ALL, and only about quarter of the genes were common between the two down-regulated sets. This led to moderate DM that was detected by mean methods but not by variance methods.

Many studies have associated childhood B-ALL with inflammatory response either directly or due to the therapy-induced toxicity. A study quantified a panel of cytokines and chemokines from blood serum of children with ALL and reported pro-inflammatory status even in the absence of apparent infection [55]. Another study highlighted cytokine release syndrome (CRS) as the most common therapy-induced toxicity resulting from CART-T therapy where an abundant release of cytokines leads to excessive activation of immune cells, manifesting as a wide range of symptoms including systemic inflammatory response [56].

Polymorphic interferon-gamma (IFN-γ) alleles are associated with prednisone response in childhood B-ALL, suggesting distinct effects of IFN-γ in immunosurveillance and early response to steroid therapy [57]. A study showed that natural killer cells demonstrate downregulation of activating receptors and upregulation of inhibitory receptors with impaired IFN-γ production and cytotoxicity in childhood B-ALL [58]. A more recent study presented the reaction of ALL cell lines and patient-derived xenografts to cytokines tumor necrosis factor alpha (TNF-α) and IFN-γ displayed great heterogeneity in cell death where some samples show a dose-dependent cell death by both cytokines while others show no reaction or an increased viability [59]. It is worth noting that our methods detected DV in both IFN-γ and TNF-α signaling gene sets.

MYC is a proto-oncogene transcription factor targeted by glucocorticoid (GC)-induced repression [60, 61]. The link between MYC suppression and GC-induced apoptosis in human leukemic cells was established long time ago [61]. MYC targets V1 was found significantly enriched with downregulated genes in childhood B-ALL patients with ETV6-RUNX1 fusion [62]. Similar findings were reported in [63], which showed that MYC targets were upregulated in mixed lineage leukemia or MLL-rearranged infant B-ALL but depleted in ETV6-rearranged patients. Another study examined the acquired resistance to DOT1L inhibition in MLL-rearranged B-ALL cells, which leads to partial loss of MLL-fusion driven gene expression. Using GSEA on RNA-seq data, the study detected a few enriched Hallmark gene sets including MYC targets v1 [64].

Tumor growth factor-β (TGF-β) signaling induces nuclear localization of the aminoacyl-tRNA synthetase-interacting factor 2 (AIMP2) which enhances ubiquitin-dependent degradation of the FUSE-binding protein (FBP), a transcriptional activator of MYC [65]. A retrospective study analyzed gene expression dataset of GC-resistant and GC-sensitive B-ALL infants with MLL-rearrangements and detected a gene module enriched with aminoacyl-tRNA synthetases (ARSs) in the GC-resistant phenotype [66]. The study suggested a failure to repress MYC and initiate GC-induced apoptosis due to increased cellular activity of TGF-β-induced ARSs interacting factors in the GC-resistant phenotype. Another study showed that the TGF-β/SMAD signaling pathway is an important mediator of natural killer cell dysfunction in childhood B-ALL, contributing to evasion of innate immune surveillance and resistance to anti-leukemic cytotoxicity [58]. Another study compared good and poor responders to prednisolone in childhood B-ALL with regard to the potential role of genes EMP1, CASP1, and NLRP3 in prednisolone response [67]. The study showed that genes positively correlated with EMP1 were associated with multiple signaling pathways, including TGF-β and TNF-α signaling pathway.

Tumor necrosis factor-α (TNF-α) is a proinflammatory cytokine that initiates immune response in part by activating the transcription factor NF-κB. NF-κB was identified as one of the three transcription factors targeted by GC-induced repression which leads to apoptosis in hematological cells [60]. Many studies have shown the interaction between GC receptor and the p65 subunit of NF-κB [68–72]. Tissing et al. identified 51 transcriptionally regulated genes in childhood ALL cell lines at 8 hours post-exposure to prednisolone, including 11 genes related to NF-κB signaling [73]. In vitro studies have shown that NF-κB inhibition induces apoptosis in leukemic stem cells [74]. TNF-α treatment applied to HeLaS3 and COS1 human cell lines led to NF-κB-mediated regulation of two protein isoforms of the GC receptor where the accumulation of the nuclear localized β isoform over the cytoplasmic α form was correlated with GC resistance [75]. However, NF-κB inhibition acts on normal cells and induce inflammatory molecules, particularly TNF-α, increasing sensitivity to cell death signals [76]. TNF-α induces NF-κB-dependent and NF-κB-independent survival signaling, hence promoting proliferation of leukemia cells [77, 78]. A study showed that the inhibition of TNF-α enhanced NF-κB inhibition-induced apoptosis in leukemia cells while protecting healthy hematopoietic and other tissue cells [79].

The unfolded protein response (UPR) is an evolutionarily adaptive response that detects the accumulation of unfolded or misfolded proteins and adjusts the protein folding ability of the endoplasmic reticulum (ER) [80]. Studies have suggested that UPR contributes to chemotherapy resistance in B-ALL and lymphoma cells [81, 82]. UPR activates due to ER stress caused by altered cell protein homeostasis, and the activation is mediated by three main sensors: PERK, ATF6α, and IRE1α [83]. These three sensors are kept inactive through binding to the stress sensor 78-kDa glucose-regulated protein (GRP78) [84]. In response to stimuli, GRP78 binds with a higher affinity to unfolded or misfolded proteins within the ER, thereby detach from the three ER-stress sensors [85]. ER stress induction triggers IRE1α to induce a conformational change that activates the RNase which leads to a general downregulation of the ER load via unconventional splicing of the X-box binding protein 1 (XBP1) mRNA [83]. It was shown that IRE1α, XBP1, and GRP78 were upregulated in B-ALL patients at diagnosis and at relapse, and high levels of these proteins predicted a poor outcome [86, 87]. For the Philadelphia chromosome positive (Ph+) pre-B-ALL cells, XBP1 was demethylated and upregulated under the BCR-ABL1 subtype [86]. GRP78 was abundantly expressed and contributed to chemotherapy resistance of leukemic B-cell precursors [87]. These observations have advocated the use of ER stressors as potential drugs for the treatment of B-ALL [83, 86–89].

The promoter region of the estrogen receptor gene is aberrantly methylated in 86% of human hematopoietic tumors, including 8 of 9 pediatric ALL [90]. Gallagher et al. showed that the estrogen-related receptor-β (ESRRB) transcription factor cooperates with the GC receptor to mediate the GC gene expression signature in mouse and human ALL cells [91]. Bardini et al. [92] investigated the effects of bromodomain and extra-terminal (BET) inhabitation in a preclinical mouse model of MLL-rearranged infant ALL and reported in vivo impairment of the leukemic engraftment of patient-derived primary samples in mice. The study also reported that the treatment sensitizes GC-resistant MLL-rearranged cell lines to prednisolone in vitro. Transcriptional change associated with the treatment was enriched in the xenobiotic metabolism and estrogen response (early and late) [92].

Notch signaling can play oncogenic or tumor suppressor roles, depending on cell type [93]. A study highlighted the role of Notch signaling in the stromal cell-dependent protection of B-ALL cells from chemotherapy-induced apoptosis where the inhabitation of Notch signaling in the presence of hydrocortisone dramatically decreased the level of overall live B-ALL cells co-cultured with human bone marrow mesenchymal stromal cells [94]. Additional results supported the enhanced chemo-sensitivity of B-ALL cells via the inhibition of Notch signaling [95]. Multiple Notch signaling pathway genes contain CpG islands in their promoter regions and showed higher and more frequent methylation than normal controls. Among others, the Notch target gene Hes5 was hypermethylated in B-ALL and T-ALL cells compared to normal controls with more frequent and greater methylation in B-ALL compared to T-ALL [96]. Reversing the transcriptional silencing of Hes5 due to promoter methylation led to growth arrest and apoptosis in B-ALL cells [96].

Synthetic GCs such as prednisolone are xenobiotics that could have harmful toxic effects if inadequately metabolized by the cytochrome P450 enzymes (CYP) [97]. Clearing of prednisolone from the body occurs primarily by the hepatic metabolism [98], which is part of the xenobiotic metabolism. Studies have associated host genetic polymorphism of xenobiotic metabolizing enzymes with host susceptibility and response to drugs. Krajinovic et al. investigated if CYP2E1, NQO1, and MPO variants modify risk in childhood ALL (predominantly B-ALL) [99] and reported that CYP2E1 and NQO1 variants significantly contributed to the risk, but not MPO variant alone. Interestingly, CYP2E1 and NQO1 are members of the xenobiotic metabolism gene set, but not MPO. The same group showed that variants in CYP1A1 (xenobiotic metabolism gene set member), among other genes, are significant predictors of childhood ALL risk [100], and variants of CYP1A1 and NQO1 were associated with poor prognosis in pediatric ALL [101]. Another study reported risk association for a CYP2E1 variant in both ALL and AML [102]. These studies provide support for associating the xenobiotic metabolism gene set with prednisolone resistance.

### Colon polyps dataset.

The potentially malignant sessile serrated adenoma/polyps (SSP) account for 15–30% of colon cancers [103]. These polyps have been distinguished from the benign hyperplastic polyps (HP) by their endoscopic appearance (larger, flat and hypermucinous) and histologic characteristics (dilated crypts, horizontal crypts, and boot shaped deformities) [104–107]. Similarities between SSP and HP often led to low consensus in classification, especially for borderline phenotypes. Studies have shown that considerable proportions of samples that were classified as HPs were probably SSPs [108, 109]. We hypothesized that SSP or HP phenotype heterogeneity will lead to molecular variability in relevant gene sets that could be detected using our proposed DV methods. Therefore, we focus on the interpretation of gene sets detected by the majority of methods that detect DV by presenting studies from literature that link them to homeostasis of the intestinal epithelium, tumor initiation and progression, or colon cancer. We repeated the steps performed for the childhood B-ALL datasets to detect Hallmark gene sets that show significant DV by the majority of the four used methods. Our DV methods detected five gene sets (Figure 3), including three related to canonical oncogenic pathways: Apoptosis, epithelial mesenchymal transition (EMT), and reactive oxygen species. Two additional gene sets were detected: Wnt-β-catenin signaling and spermatogenesis. Detecting significant DV in the three oncogenic gene sets reflect the fact that a subset of SSPs may progress to carcinoma while the rest remain in dormant state. Balanced cell death through apoptosis is necessary to maintain the homeostasis of colorectal mucosa, and defective regulation of apoptosis is one factor that lead to the progression of an adenoma to carcinoma [110]. The EMT process is necessary to maintain the homeostasis in adult tissue and involves epithelial cells acquiring a mesenchymal phenotype with increased motility, promoting the spread of tumor cells [111]. Inflammation-activated human mesenchymal stem cells (MSCs) promote the EMT process and progression of colorectal cancer (CRC) through the CCL5/β-catenin/Slug pathway [112]. The neural cell adhesion immunoglobin-like molecule L1CAM is a target of the Wnt-β-catenin signaling that promote stemness [113], leading to the elevated transcription of markers of intestinal and colonic epithelial stem cells [114, 115]. Detection the Wnt-β-catenin signaling by our methods supports the MSC-mediated changes in the EMT process.

Wnt-β-catenin signaling is essential for the homeostasis of the intestinal epithelium [116] and plays a pivotal role in tumor initiation and development through involvement in cell survival, proliferation, apoptosis and autophagy [117]. Modifications and degradation of β-catenin are key events in the Wnt signaling pathway and the development and progression of colon cancer [118]. Previous studies support the detection of the Wnt-β-catenin signaling using our DV methods by highlighting the wide variability in β-catenin leading to conflicting results on Wnt signaling activation in SSPs [119]. Detecting significant DV in the spermatogenesis gene set is an indirect result for the defective regulation in the Wnt-β-catenin signaling which plays a major role in normal reproductive tract development, male germ cell development, and spermatogenesis [120–124].

Reactive oxygen species (ROS) play a critical role in tumorigenesis by regulating signaling pathways that drive proliferation, evasion of apoptosis, migration, and malignant progression [125, 126]. ROS promote the EMT process [127, 128], which has also been detected by our DV methods. ROS production and NF-κB activation triggered by Ras-related C3 botulinum toxin substrate 1 is critical for the initiation of CRC [129]. Zhou et al. used single-cell RNA-sequencing to explore the early molecular alterations underlying the serrated neoplasia pathway towards CRC and suggested that upregulation of SERPINB6 in the epithelia of serrated lesions can promote serrated tumorigenesis via modulating ROS levels [130]. Kostic et al. examined the influence of gut microbiota on signaling pathways in colorectal serrated neoplasia and highlighted Fusobacterium Nucleatum, a bacterium that increases the production of ROS, possibly by selectively recruiting myeloid-derived immune cells [131], which promote carcinogenesis in CRC through oxidative metabolism [132].

## Discussion

This study demonstrates a new development for gene set analysis methods, namely the analysis of pathways variability between phenotypes with different test statistics. We employ MST-based node ranking approaches to generalize two univariate statistics into new multivariate gene sets analysis methods that detect differential sample variance or mean between two phenotypes. The study shows the ability of the new methods to detect specific alternative hypotheses through simulations, as well as real gene expression data.

Our simulation results show that all four methods and MST order options (K=1,2, and 3) control Type I error rate at the specified significant level (α=0.05). All methods demonstrated the desired behavior by detecting specific alternative hypotheses (DM: $\mu_{X}\neq \mu_{Y}$ or DV: $\sigma_{X}\neq \sigma_{Y}$). The detection power under different parameter settings (Additional File 1) reveals a few patterns: First, increased sample size leads to increased detection power as expected in nonparametric methods. Second, increased intergene correlation slightly decreases detection power against DV but not DM. This is expected since a uniform increase in intergene correlation reduces variability between genes. Third, all four methods perform similarly with RKS showing slightly lower detection power than RMD, RCVM, and RAD for small sample size. This minor difference becomes negligible when sample size increases.

**Figure 4.**
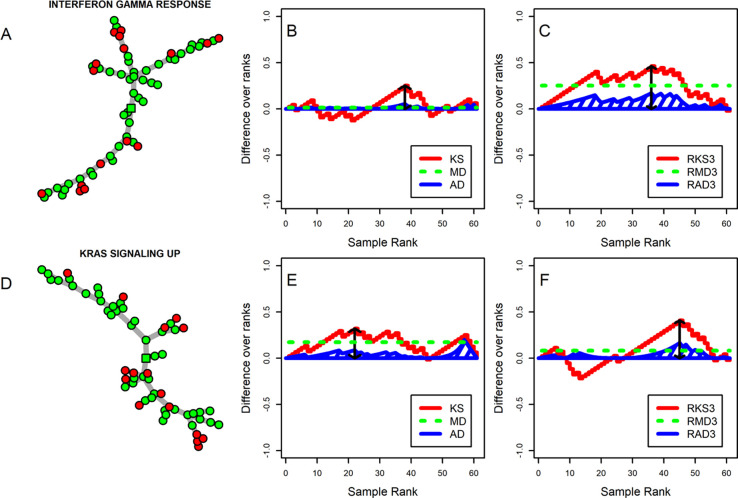
Estimated deviation by different statistics in the Interferon Gamma Response (panels A, B, and C) and the KRAS Signaling Up (panels D, E, and F) gene sets in the childhood B-ALL dataset: Minimum Spanning Tree graph (A and D), estimated deviation between CDFs of two groups of HDP-ranked samples in the MST (B and E), and estimated deviation between CDFs of two groups of radial-ranked samples in the MST (C and F).

Two real datasets showed that the Hallmark gene sets detected by the majority of differential variance methods are associated with the two compared phenotypes with support provided in literature. The four methods showed agreements and disagreements in the returned significance levels since they put emphasis on different regions of the deviation between two empirical CDFs of two groups of ranked samples in the MST. We demonstrate this behavior in the Interferon Gamma Response and KRAS Signaling Up gene sets using the childhood B-ALL dataset. Figure 4 shows the MSTs (A and D), estimated deviation between CDFs of two phenotypes when samples are HDP-ranked (panels B and D) or radial-ranked (panels C and F) in the MST. The curves of AD (and RAD) and MD (and RMD) were respectively normalized by $n_{1}\times n_{2}$ and $N=n_{1}+n_{2}$ to scale them to a similar range to the KS (and RKS) curve. For the Interferon Gamma Response, we detected significant DV (Figure 4C) but not DM (Figure 4B) using all four methods. The RKS statistic indicated by the maximum value of the RKS curve (marked by the black arrow), the RMD statistic indicated by the dashed green line, and the RAD statistic indicated by the area under the blue curve are larger in panel C as compared to panel B. For the KRAS Signaling Up, only RKS detects significant DV, while AD and MD detect significant DM. Observations from gene sets show that large differences between two phenotypes mostly lead to agreement among the four methods, while small differences sometimes lead to disagreement between methods.

## Conclusions

We have proposed novel gene set analysis methods designed to detect differential sample mean or variance between groups in gene sets. We characterized these methods using simulated gene expression data and demonstrated the usefulness of methods designed to detect differential sample variance in providing biological interpretations when biologically relevant but heterogeneous changes between groups are prevalent in specific signaling pathways. We demonstrated the ability of these methods to detect heterogeneous changes in meaningful signaling pathways associated with compared groups in two real datasets: 1) prednisolone-resistant vs. prednisolone-sensitive children diagnosed with B-ALL, and 2) benign hyperplastic polyps vs. potentially malignant sessile serrated adenoma/polyps. Such changes render some signaling pathways undetectable by most GSA methods designed to detect differential mean. The study presents two novel multivariate generalizations of the Cramer-Von Mises and Anderson-Darling tests based on sample ranking schemes in the minimum spanning tree. Software implementation of the two new methods in addition to two methods developed earlier is available from Bioconductor package GSAR. The four methods complement each other by using test statistics that put emphasis on distinct patterns of change depending on the distribution of samples in a minimum spanning tree. Using a majority vote rule or a p-value combination scheme adds additional confidence in results. The available methods are easily applicable not only to transcriptomics and proteomics datasets, but also for any omics datasets in a normalized matrix form with available collection of gene sets.

## Figures and Tables

**Table 1.** Estimated Type I error rate from simulated data at sample size *N*=40 and gene set size *p*=60 (*α*=0.05)

**Method Placement**			
RKS1	RMD1	RCVM1	RAD1
RKS2	RMD2	RCVM2	RAD2
RKS3	RMD3	RCVM3	RAD3
KS	MD	CVM	AD
**Type I Error Rate**			
0.033	0.046	0.048	0.048
0.030	0.043	0.045	0.044
0.032	0.046	0.047	0.049
0.033	0.033	0.047	0.047

## Data Availability

All the datasets used in this study are publicly available and accessible from the Gene Expression Omnibus repository under accession numbers GSE655, GSE656, and GSE76987. All the performed methods in this study are available publicly with reference manual, code examples, and vignette documentation in package GSAR from the Bioconductor repository https://bioconductor.org/packages/release/bioc/html/GSAR.html.

## List of abbreviations

AD: Anderson-Darling
B-ALL: B-lineage acute lymphoblastic leukemia
CDF: Cumulative distribution function
CVM: Cramer-Von Mises
DM: Differential sample mean
DV: Differential sample variance
GEO: Gene expression omnibus
GSA: Gene set analysis
GSAR: Gene set analysis in R
GSEA: Gene set enrichment analysis
HDP: High directed preorder
HP: Hyperplastic polyps
KS: Kolmogorov-Smirnov
MD: Mean deviation
MSigDB: Molecular signature database
MST: Minimum spanning tree
RAD: Radial Anderson-Darling
RCVM: Radial Cramer-Von Mises
RKS: Radial Kolmogorov-Smirnov
RMA: Robust multi-array average
RMD: Radial mean deviation
SSP: Sessile serrated adenoma/polyps
